# Cytokines in PD-1 immune checkpoint inhibitor adverse events and implications for the treatment of uveitis

**DOI:** 10.1186/s12886-024-03575-7

**Published:** 2024-07-29

**Authors:** Aaron C. Brown, José Quiroz, Devayu A. Parikh, Yafeng Li, Lukas Ritzer, Richard Rosen, Avnish Deobhakta

**Affiliations:** 1 Department of Ophthalmology, New York Eye and Ear Infirmary of Mount Sinai, 310 E 14th Street, New York, NY 10003 USA; 2 Department of Ophthalmology, Icahn School of Medicine at Mount Sinai, New York, NY USA; 3 Department of Ophthalmology, Massachusetts Eye and Ear, Harvard Medical School, Boston, MA USA; 4 Einhorn Clinical Research Center, New York Eye and Ear Infirmary of Mount Sinai, New York, NY USA

**Keywords:** Uveitis, Immune Checkpoint Inhibitors, Drug Therapy

## Abstract

Immune checkpoint inhibitors (ICI) such as Programmed cell Death 1 (PD-1) inhibitors have improved cancer treatment by enhancing the immune system’s ability to target malignant cells. Their use is associated with immune-related adverse events (irAEs), including uveitis. The profile of pro-inflammatory cytokines underlying Anti-PD-1-induced uveitis shares significant overlap with that of non-infectious uveitis. Current corticosteroid treatments for uveitis while effective are fraught with vision threatening side effects. The cytokine profile in ICI-related uveitis has a large overlap with that of noninfectious uveitis, this overlap strongly supports the potential for therapy that activates the PD-1 axis in the eye to treat uveitis. Indeed, ICI related uveitis often resolves with cessation of the ICI, restoring the endogenous PD-1 axis. The potential benefit of targeting many pro-inflammatory cytokines via local PD-1 axis activation is mitigating ocular inflammation while minimizing adverse effects.

## Introduction

Immune checkpoint inhibitors (ICI) have revolutionized cancer treatment by facilitating the immune system’s ability to target malignant cells. [1, 2] Inhibition of immune checkpoints such as Programmed cell Death protein 1 (PD-1) and Cytotoxic T Lymphocyte Antigen-4 (CTLA-4) increase immune recognition in melanoma, lung cancer, and some lymphomas. [3] However, their use has been linked to the development of secondary inflammation, termed immune related adverse events (irAEs), including uveitis. [2, 4, 5]

Uveitis is a multifaceted ocular inflammatory disease, involving a complex network of molecular signaling pathways. Cytokines are pivotal mediators of inflammation and assume a central role in orchestrating the immune response within the ocular microenvironment. Infection, injury, or autoimmune/inflammatory diseases and drug-induced inflammation can lead to uveitis. Key cytokines associated in uveitis include interleukins (IL), specifically IL-6 and IL-17, tumor necrosis factor alpha (TNF-α), and interferon gamma (IFN-γ), each exhibiting distinct effects on the pathogenesis of uveitis. [6–9]

This review focuses on the role of cytokines in uveitis, with an emphasis on uveitis induced by immune checkpoint inhibitors. Understanding the cytokine profiles associated with ICI-induced uveitis not only provides insights into the mechanisms of this adverse event but also sheds light on potential unique therapeutic strategies applicable to multiple etiologies of uveitis.

### ICI Therapy and irAEs

Immune checkpoints are components of regulatory pathways in the immune system that help to maintain self-tolerance, averting autoimmunity. The interaction between immune checkpoint ligands on host cells and the receptors expressed on T Cells prevents the immune cell from entering an active state to mount an immune response against the host cell. [1] The PD-1 axis is an excellent example of immune cell regulation. PD-1 is a surface expressed receptor found on T cells, B cells, dendritic and NK cells. [10–12] Its ligand, Programmed cell death ligand 1 (PD-L1), is expressed in numerous cell types located throughout target tissues including vascular endothelial cells, mesenchymal stem cells, pancreatic islets, neurons, keratinocytes, placental tissue. Germane to this review is the presence of PD-L1 on corneal epithelial cells and retinal pigmented epithelial cells . [10, 13–16] When a T Cell major histocompatibility complex (MHC) binds to a host antigen in peripheral tissue, the presentation of PD-L1 on the host binds the PD-1 receptor, resulting in a differentiation into a regulatory T cell (Treg), a cell line responsible for promoting self-tolerance, or induction of a state of anergy (“exhaustion”) whereby it cannot be stimulated to proliferate nor mount an inflammatory response. [11, 17] Notably, some cancers are able to exploit the PD-1/PD-L1 system by expressing PD-L1 on the cell surface or even secrete soluble PD-L1 to elude the host immune system. By way of comparison, the CTLA-4 receptor-ligand interaction occurs primarily in secondary lymphoid organs, representing an earlier stage of T cell activation. [18]

**Fig. 1:**
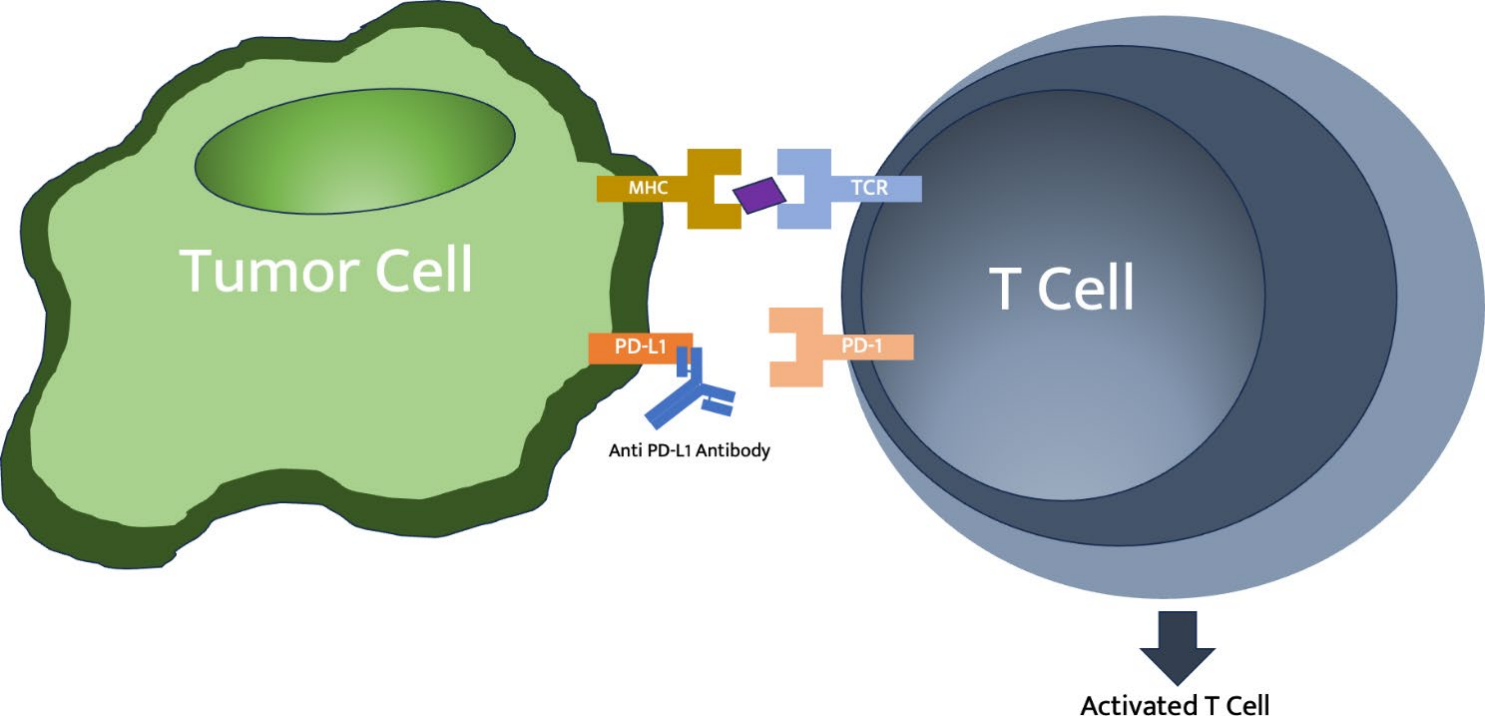
The PD-1 receptor-ligand interaction prevents T Cell activation. ICI prevent this interaction which leads to activation of T Cells. Original Image

Immune checkpoint inhibitor therapy has been effective in treating a growing number of malignancies by counteracting the evasion of cancers from host immune surveillance. [10–12] ICIs are monoclonal antibodies that directly interrupt the interaction between PD-1 and PD-L1, enabling the immune system to act against tumor cells expressing immune checkpoint ligands on the cell surface. (Fig. 1) ICI therapy is effective against a myriad of cancers and is a powerful tool against cancers that are resistant to the typical first line chemotherapies. Numerous monoclonal antibodies have been FDA approved for treatment of metastatic melanoma, small cell lung cancer, renal cell carcinoma and others. [3] Approved anti-PD-1 monoclonal antibody (mAb) therapies include pembrolizumab (Keytruda), Nivolumab (Opdivo), and Cemiplimab-rwlc (Libtayo). [3] Anti-PD-L1 mAb therapies include Atezolizumab (Tecentriq), Durvalumab (Imfinzi), and Avelumab (Bavencio). [3]

The unfortunate but expected effect of inhibiting the immune checkpoint system is a reduction of self-tolerance and a rise in autoimmune activity by T Cells, which are clinically responsible for inflammatory and autoimmune disease states. Neuropathies, anemias, thrombocytopenia, autoimmune pancreatitis, and uveitis are immune-related adverse events (irAEs) associated with ICI therapy.

The manifestation of robust anti-self activity is a specific indication of cytotoxicity of neoplastic cell therapy. A decrease in self-tolerance, which is often detectable clinically, may provide a surrogate indicator of the efficacy of the ICI therapy and response to tumor cell recognition. Thus, the presence and level of activity of irAEs provides a proxy for the effectiveness of the antitumor therapy. Recently, studies have consistently demonstrated improved survival outcomes as patients experience an increasing number of irAEs. [1, 19, 20] The presentation of irAEs are variable, ranging from a mild dermatitis to life threatening heart failure. [19, 21] Ocular irAEs include dry eye, corneal decompensation, uveitis, ocular myasthenia, and optic neuropathy . [22–24] Recent literature has identified paraneoplastic forms of carcinoma associated retinopathy (CAR), melanoma associated retinopathy (MAR) and Acute Exudative Polymorphous Vitelliform Maculopathy (pAEPVM) in association with CTLA-4 and PD-1 inhibition. [25–28] Cases of pre-existing paraneoplastic retinopathies have been shown to rapidly worsen after PD-1 inhibition. [29, 30] While irAEs may be an encouraging sign for cancer treatment, they can be associated with significant morbidity and require discontinuation of therapy or treatment with systemic steroids. Though the cause of the irAE is often the blockage of a single receptor-ligand interaction (i.e., PD-1), treatment for irAEs is more complex and requires broad suppression of inflammation through systemic steroids or targeted cytokine therapy.

In particular, the etiology of ICI-Related uveitis is incompletely understood. Retinal pigment epithelial (RPE) cells natively express high levels of PD-L1, contributing to the immune privileged status of the eye and thus are an important barrier to autoimmunity. The mechanism of ICI-Related uveitis may be attributed to the reduction of self-tolerance. [15, 31] ICI related uveitis is relatively rare, occurring at a rate of 1% of patients treated on ICI over one-year. [22] Combined therapy with multiple ICIs, female gender, and metastatic melanoma may confer increased risk of uveitis. [2, 22] The uveitis is typically mild, presenting with mild to moderate anterior chamber inflammation and light sensitivity, often solely requiring corticosteroid therapy for resolution. [2, 4, 32, 33] Severe cases, however, can be vision threatening. As a specific example, the treatment of melanoma with ICI therapy can trigger a cross reactivity of normal choroidal melanocytes and malignant melanoma cells, resulting in a Vogt-Koyanagi-Harada (VKH)-like panuveitis that frequently requires the stoppage of the inciting ICI. [4] This heterologous immunity has been observed in ICI treatment of cutaneous, subcutaneous and uveal metastatic melanomas. [22, 34, 35] ICI related uveitis can be treated as idiopathic uveitis with topical, oral, intravitreal, or intravenous (IV) steroids. Though these treatments are often effective in achieving quiescence, among their numerous side effects are the acceleration of cataracts, elevated intraocular pressure, and glaucoma. [36, 37]

### Cytokines and irAEs

The mechanism underlying irAEs is a cytokine dysregulation triggered by loss of self-tolerance. Several studies have investigated the cytokine profile in patients experiencing irAEs and found that the medley of cytokines implicated varies depending on the offending cancer type. A majority of studies have been reported on patients with metastatic melanoma treated with ICI therapy. A recent study of 98 such patients, treated either with anti-PD-1 monotherapy, nivolumab or pembrolizumab, or in combination with anti-CTLA-4 therapy, ipilimumab, were assessed longitudinally for severe irAEs with cytokine bioassays. [38] In this study, 11 cytokines were elevated in metastatic melanoma patients with severe irAEs: Fractalkine, fibroblast growth factor 2 (FGF-2), interferon alpha 2 (IFN-α2), IL-12p70, IL-1a, IL-1B, IL-1RA, IL-2, and IL-13, granulocyte colony stimulating factor (G-CSF), granulocyte-macrophage colony-stimulating factor (GM-CSF). [38] Importantly, the anti-tumor efficacy of ICI therapy did not correlate with cytokine expression, suggesting that disruption of one or more of these cytokines may not impact ICI functionality. For patients treated with anti-PD-1 monotherapy, there was an association between treatment success and serum levels of IL-2, interferon gamma-induced protein 10 (IP-10, also known as C-X-C motif chemokine ligand 10 (CXCL 10)), and monocyte chemoattractant protein 4 (MCP-4, also known as chemokine ligand 13 (CCL13)). [38, 39]

ICI therapy is also widely used in small cell lung cancer. Recent studies assessing serum cytokine levels have implicated different cytokine profiles for irAEs in the setting of anti-PD-1 or anti-PD-L1 therapy for small cell lung cancer. A 2022 study found that after controlling for age, sex, pathological type and PD-L1 expression status, elevated IL-5, IFN-α, and IFN-γ were associated with a higher risk of irAEs. [40] A follow up 2023 study added to this list an array of cytokines including IL-1β, IL-2, IL-6, IL-8, IL-12, IL-17, IFN-α, IFN-γ, and TNF-α. [41] This study also found reduced clinical benefit of ICI treatment in patients who developed elevated serum IL-8. [41]

Targeting the PD-1 receptor has also shown encouraging results in the treatment of renal cell carcinoma (RCC). [42, 43] As in the previous example, the cytokine profile associated with irAEs in this cohort is somewhat unique; elevated levels of IP-10/CXCL10 is associated with the development of irAEs in patients with RCC undergoing combination or monotherapy with ICI. [44] The exact patient cohort was also tested for the pre and post treatment levels of cytokines associated with irAEs in lung cancer and melanoma, including IL-17 A, IL‐1β,IL‐6, IL‐8, monocyte chemoattractant protein 1 (MCP‐1), also known as chemokine ligand 2 (CCL2), and TNF-α. Paradoxically, no significant increases in these levels were detected. Additionally, IL-8, associated with both irAE development and reduced efficacy of ICI treatment for small cell lung cancer, was undetectable in this patient cohort with renal cell carcinoma, further confirming the difficulty in targeting cytokines for treatment of irAEs. [44]

**Fig. 2:**
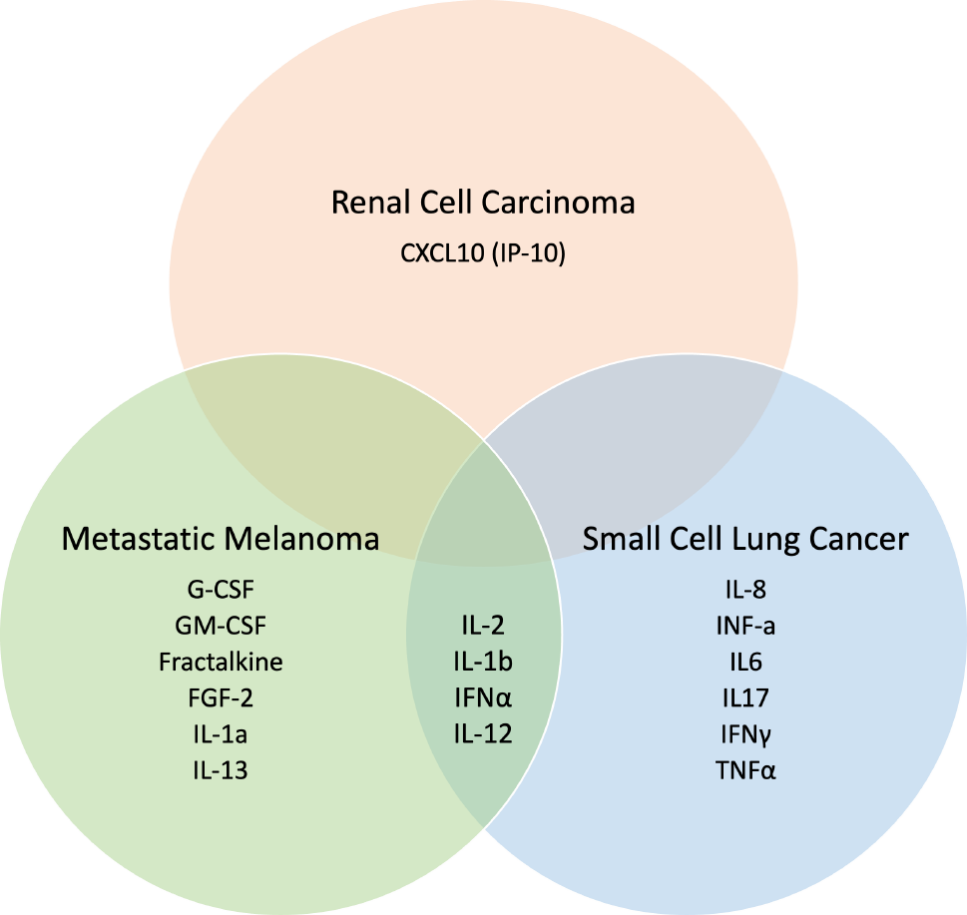
The cytokines associated with immune-related adverse events (irAEs) vary significantly depending on the specific type of cancer being treated. This Venn diagram illustrates the distinct cytokine profiles observed across different cancer types in response to Anti-PD-1 Immune Checkpoint Inhibitors. The limited overlap between cytokine signatures underscores the unique immunological landscapes associated with each cancer

The degree of morbidity of irAEs can be quite significant, requiring changing or discontinuation of ICI therapy, there exists a need to select a target to prevent or treat irAEs in this vulnerable patient population. However, these studies highlight the idiosyncratic nature of the immune system’s response to “releasing the brakes” in the setting of malignancy, making target selection more difficult (Fig. 2).

### Cytokines in uveitis

Noninfectious uveitis is an autoimmune or immune-mediated disease. Noninfectious uveitis can be associated with underlying systemic disease, such as sarcoidosis, or present without underlying disease, such as serpiginous choroiditis. Underpinning these etiologies is the activation of the host immune system resulting in damage to the eye. The pathophysiology of noninfectious uveitis is may be akin to that seen in irAE in patients receiving ICI therapy. [5, 45] Research into the pathogenesis and treatment of noninfectious uveitis offers us some insight into the cytokine dysregulation seen in irAEs.

Studies of serum cytokine levels in patients with noninfectious uveitis have consistently identified associations with elevated TNF-α, IL-6, IFN-γ and IL-17 A. [6–8, 46–50] The list of cytokines is likely much longer, including IL-8, IL-12, G-CSF, GM-CSF, MCP-1, IP-10, TNF-α and VEGF. [9] Typical treatment for uveitis involves local or systemic corticosteroid therapy to dampen the host immune response, with escalation to immunomodulatory therapy if the widespread immunosuppression of corticosteroid therapy is prolonged. Biologics targeting individual cytokines can also give excellent control of uveitis.

IL-6 is a major player in uveitis, the presence of IL-6 receptors on retinal vascular endothelial cells suggests that elevated serum levels of this cytokine can produce significant visual morbidity. [51] The STOP-Uveitis randomized clinical trial compared two strengths of an anti-IL-6R antibody, tocilizumab, in patients with non-infectious uveitis. This therapy demonstrated improvement in incidence and severity of ocular and systemic disease in both groups. [52] Anti-IL-6R therapy has also demonstrated benefit in treating chronic or refractory non-infectious uveitis. [53] Tocilizumab, used to quell non-infectious uveitis, has also shown benefit in treating or preventing irAEs in the setting of anti-PD-1 therapy. [54–56] Given these encouraging results from systemic therapy, local ocular treatment with intravitreal injections of IL-6 antibody has also been explored in mouse models. [57]

In addition, IL-17 A has also been implicated in uveitis. Research concerning both infectious and non-infectious uveitis has shown that serum IL-17 A levels are markedly elevated compared to controls, suggesting its involvement in ocular inflammation. [47] Additionally, IL-17 A contributes to macular edema by damaging the blood-retinal barrier through JAK1 signaling. [58] Studies on anti-IL-17 treatments in rat models have demonstrated potential in reducing uveitic inflammation, resulting in milder symptoms, delayed onset, and faster resolution. [59] Although IL-17 blockade did not completely prevent experimental autoimmune uveoretinitis (EAU), it reduced the presence of Th17 cells and decreased inflammation markers IL-6 and TNF. [59] Moreover, in mouse models of noninfectious uveitis, intraperitoneal injections of anti-IL-17 A caused a significant reduction in anterior and posterior uveitis, including a complete cessation of vasculitis. [59, 60] While these findings support the potential of IL-17 as a therapeutic target, systemic treatment with the human monoclonal antibody for IL-17 A, secukinumab (COSENTYX), has shown mixed results. Three phase III clinical trials treated used subcutaneous secukinumab as adjunctive therapy for recurrent non-infectious uveitis and did not find a statistically significant difference in rates of recurrence compared to placebo. [61] In a phase II clinical trial, Administration of IV secukinumab in chronic non-infectious uveitis was found to have higher response rates, faster response and greater rates of remission than subcutaneous administration. [62]

Notably, the TNF-α and TNF-α receptors are known mediators of ocular inflammation. In an experimental mouse model, TNF-α administration induced an autoimmune posterior uveitis [63] and there is a reciprocal attenuation of experimental autoimmune uveoretinitis (EAU) in mice treated with anti-TNFα therapy. [64] Moreover, samples of aqueous humor have been reported to have higher levels of TNF-α in patients with active noninfectious uveitis. [65] Patients with uveitis secondary to Behçet’s disease have higher serum levels of both serum TNFα and a soluble form of its receptor, tumor necrosis factor receptor 2 (TNF-R2), the latter thought to be a response to elevated TNFα. Aqueous samples of patients with active uveitis also contain elevated levels of soluble TNF-R. [49, 63] Targeting the TNF-α receptor and ligand are effective in treating non-infectious uveitis secondary to Behçet’s, sarcoidosis, birdshot chorioretinopathy, and numerous other etiologies. [66, 67] Indeed, etanercept, a synthesized TNF-R2 receptor linked to the Fc portion of an IgG1 antibody, has also shown success in the treatment of pediatric uveitis in patients with Juvenile Idiopathic Arthritis (JIA). [68]

Finally, while it is one of the most frequently identified cytokines identified in the serum of patients with active uveitis, IFN-γ has a complex and nuanced role in causing ocular inflammation. The response of the immune system to IFN-γ is sometimes contradictory, studies have shown both pro [69–71] and anti-inflammatory effects. [72, 73] For example, IFN-γ has a bidirectional effect on T helper cell 17 (Th17) pathogenicity depending on the stage of the disease process. [74] Despite this, targeting the cytokine has been shown to reduce inflammation; a mouse model demonstrated that anti-IFN-γ treatment introduced at initial stages of uveitis is protective, attenuating the disease process. During active or ongoing phases of uveitis however, IFN-γ inhibits effector Th17 cell responses. [74] Conversely, the endogenous development of anti-IFN-γ antibodies is associated with immunodeficiency that is particularly challenging to treat. [75] It appears likely that addressing elevated IFN-γ in the treatment of uveitis involves a restoration to homeostasis rather than wide spread suppression or elevation.

**Fig. 3:**
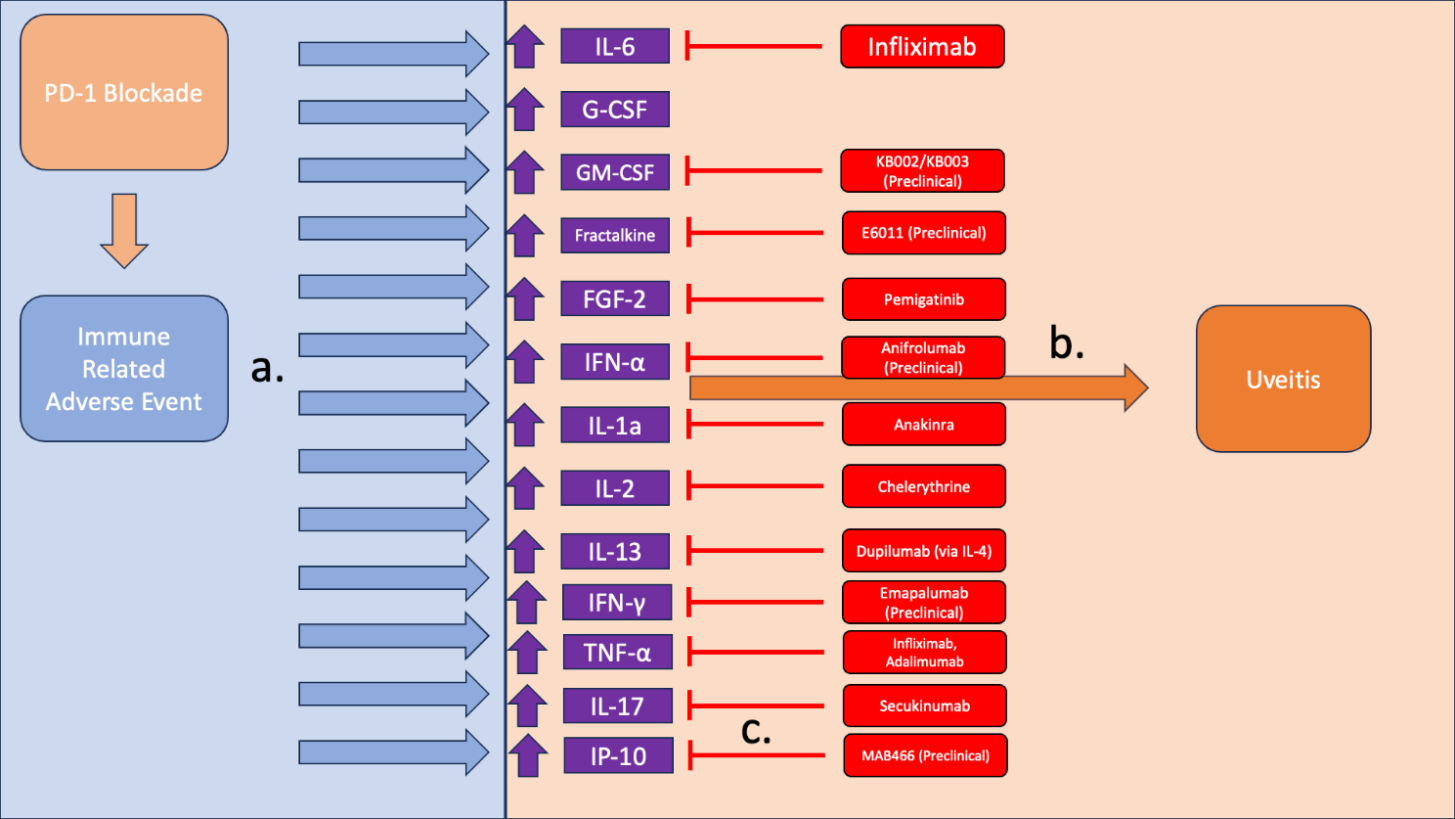
PD-1 blockade, through the use of PD-1 or PD-L1 mAbs, can trigger immune related adverse events (irAEs) through the release of proinflammatory cytokines (**a**). In the eye, elevation of this set of cytokines presents as intraocular inflammation (**b**) Inflammation secondary to cytokine release is treated with targeted therapy, typically with reduction of a single cytokine (**c**)

Figure 3 highlights that while targeting each of these cytokines can be effective, there is no panacea; patients requiring systemic immunomodulatory therapy (IMT) may need to try several different therapies before achieving sufficient suppression of inflammation. In addition, the side effect profile is not insignificant and can require discontinuation of the therapy. These challenges could be potentially addressed by a local therapy that reduces activity of pro-inflammatory cytokines.

**Fig. 4:**
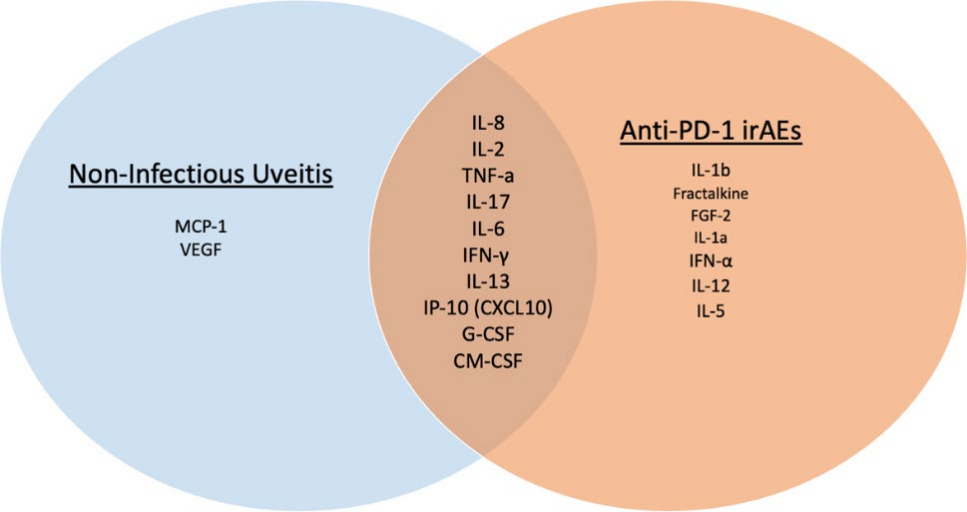
The cytokine profiles elevated in Non-Infectious Uveitis and Anti-PD-1 irAE Uveitis demonstrate a notable degree of overlap

### A PD-1 opportunity

The presence of uveitis in patients undergoing PD-1 inhibition suggests a common link between noninfectious uveitis and irAE in the eye. As demonstrated in Fig. 4, there is significant overlap between cytokines responsible for non-infectious uveitis and those elevated in anti-PD-1 associated irAEs. [55, 61, 76–85] Notably, 10 of the 12 cytokines associated with uveitis are also elevated in the setting of anti-PD-1 therapy. The large overlap in cytokine profile suggests that the PD-1 axis may play a role in the development and propagation of uveitis. Targeting individual cytokines from this cytokine profile (Fig. 4) such as IL-6 has also been shown to reduce inflammation. For instance, tocilizumab reduces levels of IL-6, etanercept reduces levels of TNF-α, and secukinumab reduces IL-17. Direct agonism of the PD-1 axis could potentially achieve this same reduction in pro-inflammatory cytokines with the additional benefit of reducing all the other cytokines whose elevation is associated with irAE uveitis.

**Fig. 5:**
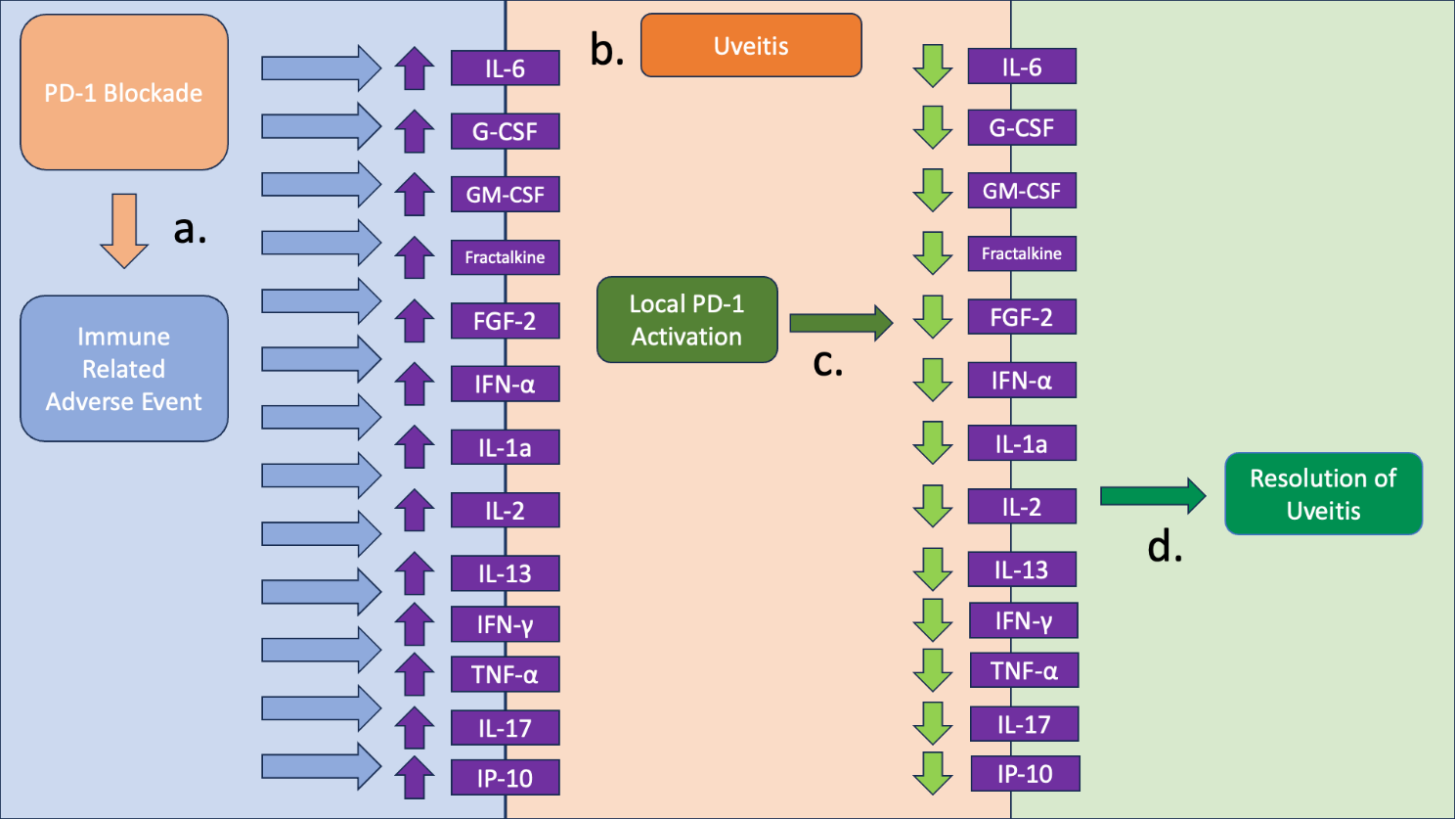
PD-1 blockade, through the use of PD-1 or PD-L1 mAbs, can trigger immune related adverse events (irAEs) through the release of proinflammatory cytokines (**a**). In the eye, elevation of this set of cytokines presents as intraocular inflammation (**b**), with anterior, intermediate, posterior uveitis or any combination thereof. Intravitreal delivery of PD-1 agonist (**c**) could restore the PD-1 axis with a return of cytokines to homeostatic levels. With the abatement of inflammatory signaling, uveitis could in turn resolve (**d**)

Thus, if blocking the PD-1 axis causes a sufficient elevation in proinflammatory cytokines to cause uveitis, it is possible that systemic or even local *agonism* of that axis would normalize the levels of these inflammatory cytokines with resolution of uveitis. Therefore, in uveitis not caused by anti-PD-1 therapy, locally fortifying the PD-1 axis may cause analogous reduction of the implicated cytokines, as demonstrated in Fig. 5.

### Corticosteroid is an imperfect treatment for uveitis

Corticosteroid therapies are effectively the standard of care for all types of ocular inflammation, representing one out of every four eye drop prescriptions in the United States. [86] Steroids form the foundation of the treatment of uveitis and are given in every manner conceivable: topical, peribulbar, intracameral, subconjunctival, sub-Tenon’s, suprachoroidal, intravitreal, retrobulbar and oral. The anti-inflammatory effect of steroids is achieved at the transcriptional level. The cholesterol-rich chemical structure of steroids enables transition through the cell membrane, to reach the nucleus where interaction with the glucocorticoid receptor is achieved. The glucocorticoid receptor has a robust effect on DNA transcription affecting a multitude of pathways; one of the most significant being the inhibition of the proinflammatory nuclear factor kappa B (NF-kB) transcription factor. Steroids cause a deacetylation of histones activated by NF-kB, closing off the DNA primers responsible for promoting transcription of inflammatory cytokines. [87] Specifically, inhibition of NF-kB by steroids blocks induction of the genes for IFN-γ, [88] TNF-α, IL-1β, IL-6, IL-8, IL-12p40, [89] IL-17, [90] among many others. The blockade of these cytokines, implicated in uveitis, establishes the critical role steroids play in the current anti-inflammatory treatment paradigm.

However, the direct access to nuclear activity also results in undesirable effects in the eye. While virtually every orbital or periorbital tissue can be negatively impacted by the use of steroids, the most common adverse effects are ocular hypertension and cataract, occurring in greater than 10% of treated patients. [37]

Steroid-induced elevation in intraocular pressure (IOP) is a common and particularly pernicious side effect of steroid treatment, up to 36% of the general population has an increase of 5 mm Hg or more in response to topical steroid treatment. This percentage increases to up to 92% of those with a history of primary open angle glaucoma (POAG). [91–94] Because uveitis often requires sustained immunosuppression, patients may need steroid treatment for long periods of time further increasing the risk of pressure elevation coupled to inflammatory damage of the trabecular meshwork. This results in damage to the optic nerve at a reported incidence of 8–35%.[95, [96]] These side effects can be an especially challenging problem in treating children with uveitis, who develop elevated IOP at a similar rate as adults [97] but require filtration surgery to control IOP at much higher rates, cited around 1/3 of all children with uveitis. [95, 97, 98] Among these children, vision threatening complications after filtration surgery occur at a rate of 10% over 5 years. [99]

Steroid-induced cataract progression is another challenging adverse effect in the uveitis population. Patients with uveitis have a baseline increased risk of cataract formation that grows in a dose-dependent manner with use of topical steroids to 55%, with just twice per day administration. [100, 101] Over a 7 year period, any topical corticosteroid use is associated with three-times increased risk of cataract surgery. [101, 102] Patients with uveitis that undergo cataract surgery have worse postoperative visual acuity, higher rates of glaucoma, higher rates of post-operative cystoid macular edema (CME), posterior capsular opacification, recurrent inflammation and epiretinal membrane formation. [50, 100, 102–106] Because of the complexity of these cases, there is also an increased risk of intraocular lens (IOL) dislocation and decentration. [107, 108]

The adverse side effects of steroid treatment are significant, and even patients on immunomodulatory therapy often require intermittent steroid pulses to control the disease. Moreover, even when such side-effects are deemed clinically acceptable, patients with irAEs may be steroid refractory in 5–25% of cases [109–111] Thus, while the steroid immunosuppression can alter the disease course, it is not a proverbial off switch; but more akin to a carpet bomb that alters transcription with innumerable negative downstream effects. Optimal treatment of uveitis focusing on a target which can restore the homeostasis of cytokines without damaging ocular structures is a clear unmet need.

### PD-1 activation as steroid sparing therapy

Modulating the PD-1/PD-L1 axis may be the proverbial inflammatory off-switch needed for uveitis. The cytokine profile in PD-1 blockade-related uveitis has a large overlap with that of noninfectious uveitis, this overlap strongly supports the potential for therapy that activates the PD-1 axis in the eye to treat uveitis. Indeed, ICI related uveitis often resolves with cessation of the ICI, restoring the endogenous PD-1 axis. Antibody mediated paraneoplastic syndromes, such as MAR, CAR, pAEPVM, occurring after the initiation of PD-1 inhibition have also been reported to slow or improve after cessation. Furthermore, patients with active uveitis have lower serum levels of PD-L1. [25, 112–117] Activation of PD-1 receptors with an intravitreal injection of PD-L1 could cause an interruption of inflammatory cytokine release in the same way that host PD-L1 activates the previously blocked receptors after cessation of anti-PD-1 therapy. Moreover, as the PD-1 receptor is only present on immune cells, PD-L1 delivered into the eye cannot interact with ocular tissues and would not cause cellular changes seen in corticosteroid therapy. In addition, while there may be a concern of T-cell anergy with PD-1 agonism, the expression of PD-L1 by tumors did not result in higher rates of infections, particularly as compared to documented rates of infections with corticosteroids. [118]

## Conclusion

This review has highlighted the critical role of cytokines in mediating immune responses in both uveitis and immune checkpoint inhibitor-induced uveitis. We have explored the limitations of current corticosteroid treatments for uveitis and discussed the potential of PD-1 agonism as a novel therapeutic strategy.

Cytokines, including IL-6, TNF-α, IFN-γ, and IL-17, play a central role in the pathogenesis of uveitis. The disruption of the PD-1 axis can trigger uveitis that is associated with these cytokines. While corticosteroids are effective in controlling uveitis, they come with significant side effects, limiting their long-term use. On the other hand, local PD-1 activation within the eye may be a promising steroid-sparing approach to uveitis therapy. Confirmation of this hypothesis will require creative clinical developments.

## Data Availability

No datasets were generated or analysed during the current study.
